# Parliament and the Professions

**Published:** 1920-05-01

**Authors:** 


### Parliament and the Professions.

### Municipal Hospital.

In reply to several questions, Dr. Addison stated that he had sanctioned a scheme supported by all parties on the Bradford Council for the establishment of a municipal hospital, which appeared to be the only practicable method of providing the institutional accommodation necessary to meet the needs of the city. Pending further legislation, which is being prepared, all similar schemes must be considered on their merits by the Ministry.

### Pensions of Naval and Military Medical Men.

Mr. Robert Young asked the Minister of Pensions the number of regular naval and military medical men employed by the Ministry who are in receipt of pensions; and how many of these receive pensions of over £500 a year.

Major Tryon replied that there are twelve regular naval or military medical men employed on a salaried basis in various parts of the country who are in receipt of pension in respect of their service; six of these officers are in receipt of pensions of £600 a year or more. They represent about 1 per cent. of the total of medical men employed.

### Territorial Medical and Veterinary Services.

Mr. Churchill stated that all Territorial officers selected for appointments as assistant directors of Medical Services of a division reside within the divisional area to which they have been appointed in order that they may be fully conversant with local conditions. Although county associations are not being asked to nominate officers for these appointments, all cases where officers have been so nominated have received sympathetic consideration. The foregoing also applies to deputy assistant directors of Veterinary Services.

### Blinded Soldiers.

Major Tryon, answering a question for the Pensions Minister, stated that approximately 1,300 soldiers discharged for total blindness have been pensioned under the Royal Warrant, and 110 have been refused pensions. The latter are men whose blindness arose from causes not connected with their military service, or was due to their own serious negligence or misconduct.

### Vaccination.

Dr. Addison stated that the approximate cost of public vaccination in England and Wales was £168,000 in the financial year 1912-13 and £112,000 in the year 1918-19.

